# Integrity and Quantity of Total Cell-Free DNA in the Diagnosis of Thyroid Cancer: Correlation with Cytological Classification

**DOI:** 10.3390/ijms18071350

**Published:** 2017-06-24

**Authors:** Francesca Salvianti, Corinna Giuliani, Luisa Petrone, Irene Mancini, Vania Vezzosi, Cinzia Pupilli, Pamela Pinzani

**Affiliations:** 1 Department of Experimental and Clinical Biomedical Sciences, Molecular and Clinical Biochemistry Unit, Careggi Hospital and University of Florence, 50134 Florence, Italy; francesca.salvianti@unifi.it (F.S.); irene.mancini@unifi.it (I.M.); 2 Department of Clinical, Experimental and Biomedical Sciences, Endocrinology Unit, Careggi Hospital and University of Florence, 50134 Florence, Italy; corinna.giuliani@tiscali.it (C.G.); luisa.petrone@aouc.unifi.it (L.P.); 3 Division of Pathological Anatomy, University of Florence, 50121 Florence, Italy; vvezzosi@unifi.it; 4 Endocrinology Unit, Santa Maria Nuova Hospital, Florence, Azienda USL Toscana Centro, 50122 Florence, Italy; cinzia.pupilli@uslcentro.toscana.it

**Keywords:** cell-free DNA, integrity index, plasma, qPCR, papillary thyroid carcinoma

## Abstract

Cell-free DNA (cfDNA) quantity and quality in plasma has been investigated as a non-invasive biomarker in cancer. Previous studies have demonstrated increased cfDNA amount and length in different types of cancer with respect to healthy controls. The present study aims to test the hypothesis that the presence of longer DNA strands circulating in plasma can be considered a biomarker for tumor presence in thyroid cancer. We adopted a quantitative real-time PCR (qPCR) approach based on the quantification of two amplicons of different length (67 and 180 bp respectively) to evaluate the integrity index 180/67. Cell-free DNA quantity and integrity were higher in patients affected by nodular thyroid diseases than in healthy controls. Importantly, cfDNA integrity index was higher in patients with cytological diagnosis of thyroid carcinoma (Thy4/Thy5) than in subjects with benign nodules (Thy2). Therefore, cfDNA integrity index 180/67 is a suitable parameter for monitoring cfDNA fragmentation in thyroid cancer patients and a promising circulating biomarker in the diagnosis of thyroid nodules.

## 1. Introduction

Cancer-derived DNA in blood represents a promising biomarker for cancer diagnosis. Previous studies have demonstrated an increase of cell-free circulating DNA in different types of cancer [1] in comparison to the general population.

Even if it is well known that DNA concentration in plasma is elevated in cancer patients [1] and can be influenced by tumor characteristics [2], the hypotheses on its origin are still controversial and details on the mechanism of release are not completely disclosed [3]. Circulating free-DNA is released from apoptotic or necrotic cells, reflecting a differential DNA origin. Necrosis is common in solid malignant cancers and generates a spectrum of DNA fragments of different size, due to random digestion by DNases. In contrast, cell death in normal blood nucleated cells occurs mostly via apoptosis that generates small and uniform DNA fragments. Support for this hypothesis has been reported by several papers [2,4,5,6,7,8,9,10,11,12] and confirmed in recent studies demonstrating increased DNA length in plasma from patients with breast [13,14], prostate [15], colorectal [16,17,18] and lung cancer [19].

In addition, total cell-free DNA (cfDNA) concentration may also be altered in patients with various benign diseases such as trauma, stroke, burns, sepsis, and autoimmune diseases, thus limiting its value for diagnosis of cancer [20]. For this reason, the simple cell-free DNA quantitative analysis cannot provide the expected clinical specificity.

To this purpose, the search of qualitative alterations of DNA, such as mutations, loss of heterozygosity (LOH), microsatellite instability and epigenetic changes, were shown to improve the cancer specificity [2]. Tumor biomarkers identified in plasma of cancer patients may have a high diagnostic and prognostic value, however, the detection of these alterations is limited by the frequency of their occurrence in each tumor type and their tumor-specificity so that the development of different assays can be necessary when dealing with tumors with different mutational signatures.

Our attention is focused on fragmentation of plasma DNA. We extensively studied the characteristics of cfDNA in plasma of melanoma patients, evidencing that total quantity and integrity index could provide useful information to discriminate the tumor affected population from healthy individuals [9]. The test is based on the hypothesis that DNA fragments in plasma are longer than those of healthy individuals on account of an inefficient nuclease activity.

This manuscript studies patients affected by differentiated papillary thyroid carcinoma to test the hypothesis that the presence of longer DNA strands circulating in plasma can be considered a biomarker for tumor presence also in the case of thyroid cancer. We adopted a qPCR approach based on the quantification of two amplicons of different length (67 and 180 bp respectively) to evaluate the integrity index 180/67 whose performance had been previously evaluated in a case study composed of melanoma patients. Control subjects and subjects affected by benign pathologies have been considered as the references. Our aim is to investigate the ability of this parameter to provide diagnostic information related to the cytomorphological classification of this tumor performed on fine needle aspirates (FNA) of the nodular lesion.

## 2. Results

### 2.1. Plasma DNA Concentration and Integrity in Basal Blood Samples

**Figure 1:**
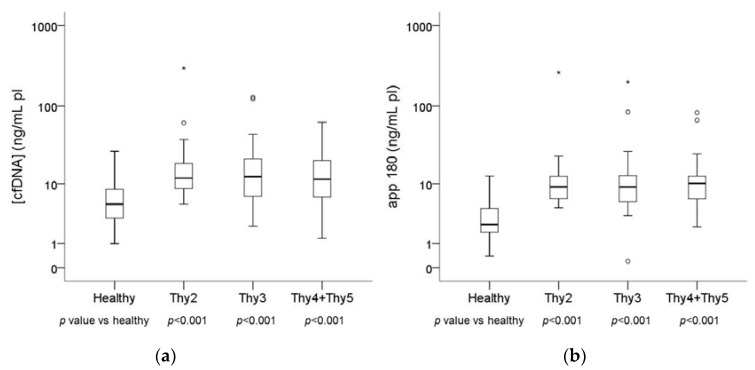
Box plots reflecting the distribution in cases (Thy2, Thy3 and Thy4/Thy5) and controls (healthy subjects) of total cfDNA quantity (**a**), and cfDNA quantity according to a qPCR assay targeting a 180 bp amplicon on the *APP* gene (**b**). Each box indicates the 25th and 75th percentiles. The horizontal line inside the box indicates the median, and the whiskers indicate the extreme measured values. Dots and stars represent outliers.

Total cfDNA was quantified by the qPCR assay targeting the 67 bp amplicon on the *APP* gene (see Methods section). Quantitative values of cfDNA concentration in plasma are reported in Table 1 and graphically represented in Figure 1a. Patients affected by nodular thyroid diseases (respectively Thy2, Thy3 and Thy4/Thy5 cytology) showed higher levels of total cfDNA than healthy individuals (*p* < 0.001).

Analogously, cfDNA quantity according to the qPCR assay targeting the 180 bp amplicon on the *APP* gene (see Methods section) was constantly higher in each patient’s cytology group (Thy2, Thy3 and Thy4/Thy5) than healthy controls with a *p* value lower than 0.001. The results are reported in Table 1 and Figure 1b.

Cell-free DNA integrity assessed by means of the integrity index 180/67 (see Methods section) was significantly higher in Thy2 (*p* = 0.01), Thy3 (*p* = 0.002) and Thy4/Thy5 (*p* < 0.001) patients than control subjects.

Subjects affected by benign thyroid nodules (Thy2) showed an integrity index significantly lower (*p* = 0.013) than that found in patients with cytological diagnosis of thyroid cancer (Thy4/Thy5).

**Figure 2:**
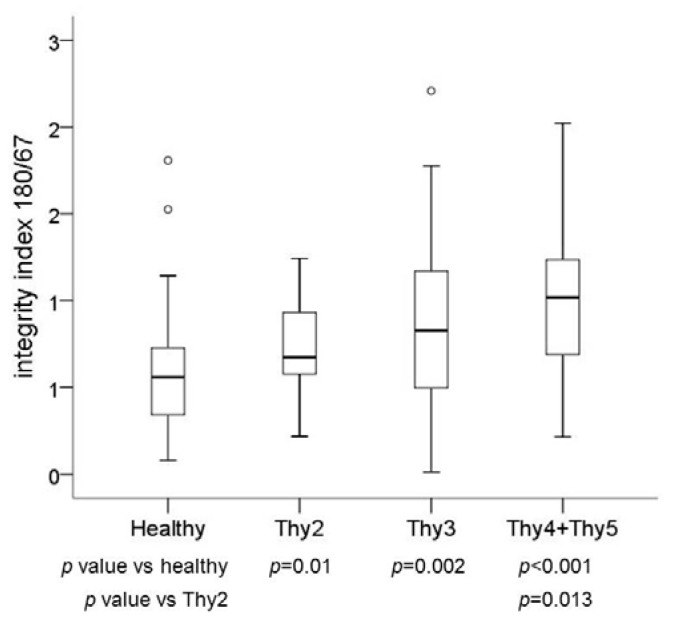
Cell-free DNA integrity in the categories of the case study: healthy subjects, Thy2, Thy3 and Thy4/Thy5. Box plots indicate the 25th and 75th percentiles. The horizontal line inside the box indicates the median, and the whiskers indicate the extreme measured values. Dots represent outliers.

The values of integrity index for each patient’s group are reported in Table 1 and Figure 2.

We did not find any significant difference in cfDNA quantity and integrity stratifying patients on the basis of sex and age.

### 2.2. cfDNA Fragments

The absolute concentration of cfDNA fragments with length ranging from 67 to 180 bp, calculated by subtracting the absolute concentration of the longer amplicon from that of the shorter one, was significantly lower in Thy4/Thy5 patients (median = −0.12, range −20.05–28.62) than subjects with Thy2 nodules (median = 3.40, range −2.24–47.94, *p* = 0.010) and healthy controls (median = 2.22, range −1.98–14.16, *p* = 0.013).

Alternatively, the percentage of cfDNA fragments between 67 and 180 bp was calculated by subtracting the absolute concentration of the longer amplicon to that of the shorter one and normalizing for total cfDNA quantity (assessed by the shorter amplicon).

Healthy controls presented about 44% of these fragments, with a significant difference with respect to Thy2 (33%, *p* = 0.010), Thy3 (17%, *p* = 0.002) and Thy4/Thy5 (−2%, *p* < 0.001) patients.

Thy2 subjects showed a higher percentage of fragments between 67 and 180 bp than Thy4/Thy5 patients (*p* = 0.013).

### 2.3. Plasma DNA Integrity in Post-Surgery Blood Samples

The specificity of the assay was demonstrated considering the variation of plasma DNA concentration and integrity from the pre-surgery value to that found after treatment, few months later. Specifically, for a group of 17 patients, an additional blood draw was taken 3–6 months after surgery and radioactive iodine treatment when appropriate.

**Figure 3:**
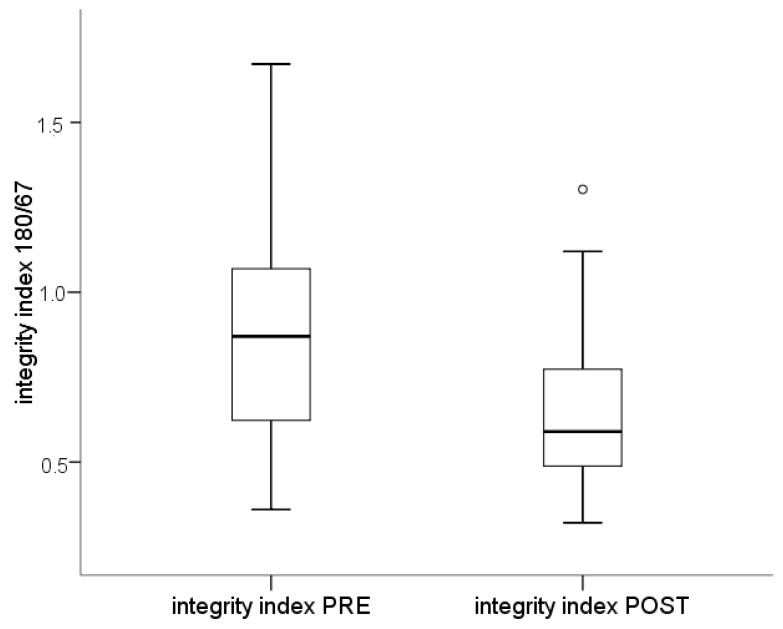
Integrity index in samples before (PRE) and after (POST) surgery. Box plots indicate the 25th and 75th percentiles. The horizontal line inside the box indicates the median, and the whiskers indicate the extreme measured values. Dots represent outliers.

While no statistical differences were evidenced, independently from the amplicon length, between pre-surgery total cell-free DNA concentration and that found at the follow-up time, the after surgery sample showed a lower integrity index (median = 0.59, range 0.36–1.67) than that taken before surgery (median = 0.87, range 0.32–1.30, *p* = 0.035, Figure 3).

After surgery, thyroid cancer patients showed a higher percentage of small (67–180 bp) DNA fragments (median = 41.02 range −30.28–67.87) in comparison to the pre-surgery condition (median = 12.99 range −67.18–64.00, *p* = 0.035).

### 2.4. ROC (Receiver Operating Characteristic) Curve Analysis

The predictive capability (i.e., diagnostic performance) of cfDNA quantity and integrity in thyroid cancer was investigated by means of the area under the ROC curve by comparing healthy subjects with patients with cytological diagnosis of thyroid carcinoma (Thy4/Thy5).

**Figure 4:**
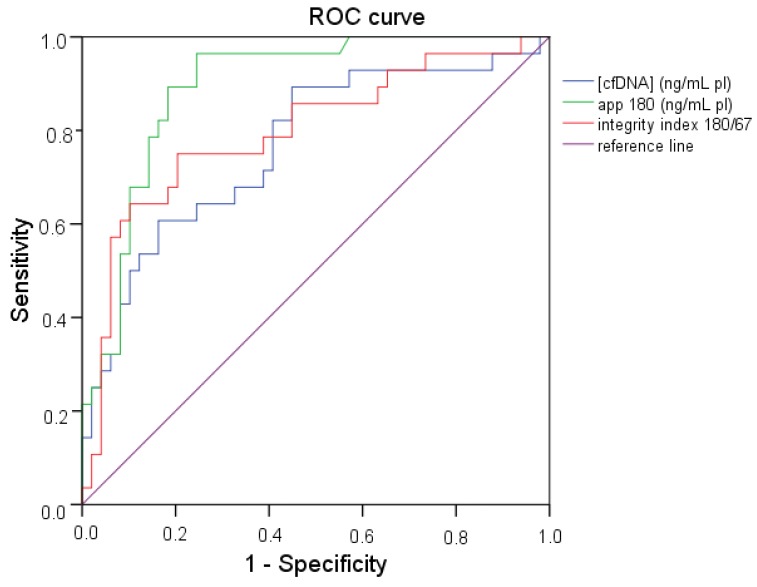
Receiver Operating Characteristic (ROC) curve of cfDNA quantity assessed by two qPCR assays targeting respectively a 67 and a 180 bp amplicon on the *APP* gene and cfDNA integrity index 180/67 in patients with cytological diagnosis of thyroid carcinoma (Thy4/Thy5) and control subjects.

All the three markers showed a good predictive capability with an area under the ROC curve (AUC) of 0.765 (*p* < 0.001), 0.982 (*p* < 0.001) and 0.796 (*p* < 0.001) for cfDNA quantity by 67 bp amplicon, cfDNA quantity by 180 bp amplicon and integrity index, respectively (Figure 4).

ROC curve analysis was also used to investigate the diagnostic performance of the markers under study by comparing Thy2 subjects with Thy4/Thy5 patients. Among the three considered parameters, only cfDNA integrity showed a good AUC (0.699) with a significant *p* value (*p* = 0.013).

## 3. Discussion

Assays performed on tumor surrogate samples are attractive mainly because of limited invasiveness of sample collection. CfDNA determination may represent an affordable way to impact on a potential diagnostic, prognostic and monitoring tool in oncology.

Notwithstanding a presumed lack of specificity of the simple estimation of the quantity and quality of total cfDNA, our previous results in melanoma patients show that, by jointly considering a panel of four biomarkers including two tumor-specific ones, the highest predictive capability was given by total cfDNA followed by integrity index 180/67 [21].

Thus, the aim of the present work was to study cfDNA quantity and the qualitative parameter integrity index 180/67 in thyroid cancer patients; to date, the latter aspect has not been investigated.

The study focused on the quantitative determination of circulating DNA by means of two qPCR assays differing in the amplicon length (67 and 180 bp respectively).

It is generally accepted that the 180 bp-fragment reflects apoptosis, which is the prevalent mechanism of cell death in normal cells, while necrosis, producing much longer DNA fragments, seems to occur more frequently in tumor cells [22,23].

A statistically significant increase could be evidenced in nodular goiter patients when compared to healthy subjects for both the absolute measurements of DNA concentration and the deriving integrity index, similarly to already published results for different types of cancer. In fact, notwithstanding a great heterogeneity in the pre-analytical and analytical steps, most of the papers based on qPCR approaches to measure DNA fragmentation in plasma report an increase of integrity index in tumor patients in comparison to the healthy population [6,7,10,13,14,15,16,17,18,19].

Besides qPCR, other approaches such as electrophoresis, electron and atomic force microscopy and, more recently, massive parallel sequencing have been adopted for the determination of cfDNA fragment size [5], providing insights into different aspects of cfDNA. However, among the above mentioned approaches qPCR represents a fast, reliable and cheap method to investigate cfDNA integrity.

The most relevant result in our study is the significant difference in cfDNA integrity index between subjects with benign nodules (Thy2) and patients with cytological diagnosis of thyroid carcinoma (Thy4/Thy5). This finding supports cfDNA integrity as a promising biomarker in the diagnosis of thyroid nodules.

Another important finding is the significant reduction of the considered integrity index evidenced 3–6 months after surgery. This result could be an indication of successful removal of the tumor, analogously to what is reported by Gang et al. [24], and demonstrates the relationship between cfDNA integrity index and tumor presence.

By evaluating the percentage of fragments in the range of length 67–180 bp, we evidenced differences between the cytological categories of the case study (Thy2, Thy3 and Thy4/Thy5) and control subjects, and a higher percentage of fragments in subjects with Thy2 nodules with respect to Thy4/Thy5 patients. Analogously, post-surgical thyroid cancer patients show a higher percentage of small DNA fragments (67–180 bp) in comparison to the pre-surgery condition. The same fragment distribution could be evidenced in our previous study on melanoma patients [9] with the smaller fragment being more abundant in control subjects with respect to melanoma and after tumor removal. These analogous results confirm that total cfDNA analysis (quantity and quality) can be considered a tumor-independent marker.

In conclusion, cfDNA integrity index 180/67 turned out to be a suitable parameter for monitoring cfDNA fragmentation in thyroid cancer patients. These data support the hypothesis that a panel of circulating biomolecular markers can be a non-invasive, valuable tool in the diagnosis of differentiated thyroid cancer and are in line with our previous findings on the detection of BRAFV600E mutation in plasma from patients with this disease [25]. As a perspective in thyroid patients, further studies using DNA integrity index to monitor patients’ outcome and the effect of therapy are advisable. Moreover, based on our previous experience in melanoma, a multimarker approach, taking into account different biomarkers related to both cfDNA quantity and quality [21], as well as markers of tumor origin, should be pursued.

## 4. Materials and Methods 

### 4.1. Patients

The presence of cfDNA circulating in plasma and its integrity features were evaluated in 97 patients (71 females and 26 males, age range 18–90 years, median 56 years) admitted to the Endocrinology Unit of Careggi Teaching Hospital because of nodular goiter between 2011 and 2015.

Patients were submitted to US-FNA according to the adopted guidelines [26] and the cytological diagnoses were made in accordance with the five diagnostic groups of the British Thyroid Association, 2007: Thy 1—non-diagnostic; Thy 2—non-neoplastic, Thy 3—follicular lesions, Thy 4—suspicious of malignancy, Thy 5—diagnostic of malignancy [27]. When more than 1 nodule was sampled by US-FNA in multinodular goiters, the worst cytology was considered.

Our case study is composed as follows: Thy2 (*n* = 25), Thy3 (*n* = 44), Thy4 (*n* = 24) and Thy5 (*n* = 4).

Seventeen patients affected by PTC were submitted to a second blood draw 3–6 months after surgery (mean 4.4 months, range 2.7–6.4 months). In this period of time, 12 patients also underwent radioactive iodine (RAI) treatment according to the adopted guidelines [28,29].

Healthy subjects were used as control populations, (*n* = 49, 26 females and 23 males, age range 25–89 years, median 53 years).

The research protocol was conducted in accordance with the guidelines in the Declaration of Helsinki and approved by the local review board “Comitato Etico Regione Toscana Sezione Area Vasta Centro” (code: CEAVC BIO 16.026, 2017); all the patients signed an informed consent.

### 4.2. DNA Extraction

Peripheral blood (5 mL) was collected in an ethylenediaminetetraacetic acid (EDTA) tube, transported within one hour to the laboratory and centrifuged twice at 4 °C for 10 min (1600 and 14,000 rcf). Plasma aliquots were stored at −80 °C before use. DNA was extracted from 2 mL of plasma, using the QIAsymphony Circulating DNA Kit (Qiagen, Hilden, Germany).

### 4.3. Plasma DNA Integrity Index 180/67 by qPCR

The quantity and integrity of the cell-free DNA circulating in plasma was evaluated by a quantitative real-time PCR (qPCR) targeting the human *APP* (Amyloid Precursor protein, chr. 21q21.2) gene (accession NM_000484). The assays were designed in a way that the forward primer and the probe were the same for all amplicons, whereas the reverse primer varied (see ref. [9] for sequences and qPCR protocol). The lengths of the amplicons selected for this study were 67 and 180 bps respectively.

Absolute quantification of the shorter amplicon (67 bp) on the gene *APP* was performed in plasma samples to accurately measure the amount of free circulating DNA per mL plasma, using primers and probe previously reported [30]. This assay was assumed to be able to measure the total amount of circulating plasma DNA, including fragments down to 67 bp of length. Quantification of DNA concentration was obtained by interpolation on an external reference curve ranging from 10 to 10^5^ pg/reaction of genomic DNA. (A DNA preparation obtained by Sigma-Aldrich was employed as the standard).

The ratio between the absolute concentration of the longer amplicon (180 bp) and the shorter one (67 bp) defined the integrity index 180/67, which was used to assess the fragmentation of cfDNA. Higher integrity index values indicate that all the cfDNA molecules are at least 180 bp in length in the *APP* gene. Lower integrity indexes mean that cfDNA contains fragments below 180 bp in the same target sequence.

### 4.4. Statistical Analysis

Statistical analysis was carried out using the SPSS statistics software package version 24 (IBM, Armonk, NY, USA). Quantitative results were evaluated by Mann–Whitney and Wilcoxon signed-rank test. *p* values lower than 0.05 were considered statistically significant. The predictive capability (i.e., diagnostic performance) of each biomarker was investigated by means of the area under the ROC (Receiver-Operating Characteristics) curve (AUC). The ROC curve measures the accuracy of biomarkers when their expression is detected on a continuous scale, displaying the relationship between sensitivity (true-positive rate, *y*-axes) and 1-specificity (false-positive rate, *x*-axes) across all possible threshold values of the considered biomarker. A useful way to summarize the overall diagnostic accuracy of the biomarker is the area under the ROC curve (AUC), the value of which is expected to be 0.5 in the absence of predictive capability, whereas it tends to be 1.00 in the case of high predictive capacity [31].

## Figures and Tables

**Table 1:** Quantitative values of cell-free DNA (cfDNA) markers in the case study.

		(cfDNA) (ng/mL pl)	App 180 (ng/mL pl)	Integrity Index 180/67
**Healthy *n* = 49**	Median	5.12	2.42	0.56
	Range	0.99–26.71	0.40–12.68	0.08–1.81
**Thy2 *n* = 25**	Median	11.88	9.03	0.67
	Range	5.10–296.52	4.55–261.04	0.22–1.24
**Thy3 *n* = 44**	Median	12.41	9.02	0.83
	Range	2.26–128.44	0.20–199.07	0.01–2.21
**Thy4 + Thy5 *n* = 28**	Median	11.47	10.15	1.02
	Range	1.31–62.60	2.19–82.65	0.22–2.02

## Abbreviations

qPCR	quantitative real-time PCR
cfDNA	cell-free DNA
